# Essential Thrombocythemia Complicated by Occlusive Thrombosis of the Abdominal Aorta

**DOI:** 10.1155/2019/9454501

**Published:** 2019-03-26

**Authors:** Jamie Geringer, Joshua Fenderson, Michael Osswald

**Affiliations:** San Antonio Uniformed Services Health Education Consortium, 3551 Roger Brooke Drive JBSA, Fort Sam Houston, San Antonio, TX 78234, USA

## Abstract

**Introduction:**

Essential thrombocythemia (ET) is a myeloproliferative neoplasm of excessive platelet production complicated by thrombohemorrhagic events. Thrombosis typically occurs in small to medium vessels; thrombosis of large vessels is rare.

**Case Presentation:**

A 75-year-old woman with ET complicated by bilateral retinal vein occlusion was evaluated for fatigue, early satiety, and unintentional weight loss. Her hypertension was well controlled, and her chronic lower extremity claudication from peripheral artery disease was stable. She reported adherence to aspirin 81 mg and hydroxyurea 1000 mg daily, and her platelets (375 × 10^9^/L) were at goal. Bone marrow biopsy was consistent with ET without progression to myelofibrosis or leukemia. CT abdomen demonstrated complete occlusion of the infrarenal aorta, extending into the common iliac arteries, with reconstitution of flow distally via collaterals. The addition of clopidogrel, for platelet inhibition, and cilostazol, for claudication, caused symptom improvement without further thrombosis or bleeding.

**Discussion:**

There are few published reports of ET complicated by aortic thrombosis. To our knowledge, this is the first report of aortic thrombosis occurring in an ET patient with normal platelet count on antiplatelet and cytoreductive therapies. There is limited evidence to guide treatment, but medical management with triple antiplatelet therapy may be effective in selected patients.

## 1. Introduction

Essential thrombocythemia (ET) is a myeloproliferative neoplasm characterized by increased platelet counts and excessive large and morphologically mature megakaryocytes in the bone marrow [1]. ET may be complicated by thrombotic and/or hemorrhagic events. Small and medium vessel thrombosis is most common and may manifest as erythromelalgia, transient ischemic attack, myocardial infarction, deep vein thrombosis, or pulmonary embolism [2]. Thrombosis of the large vessels rarely occurs, and there is limited evidence to guide management, particularly in cases without acute tissue ischemia. A small number of case reports suggest pharmacologic management alone can be successful in patients with minimally symptomatic ET-associated aortic thrombosis; however, the agents chosen and treatment rationale are quite varied [3–7].

## 2. Case Presentation

A 75-year-old African American female with a history of hypertension, hyperlipidemia, peripheral artery disease, and essential thrombocythemia, complicated by sequential bilateral central retinal vein occlusion, was seen for follow-up. She first presented to hematology in March 2015 with a history of left central retinal vein occlusion, stable claudication from peripheral arterial disease, and progressive thrombocytosis. She had quit smoking three months prior. Laboratory review revealed persistent increased platelet counts (>800 × 10^9^/L) over the previous six months. Blood smear was notable for increased large mature megakaryocytes relative to red blood cells; no left shift, leukoerythroblastosis, or dysplasia was observed. Iron studies, lactate dehydrogenase, BCR-ABL translocation, and JAK2 V617F mutation studies were unremarkable. The patient refused bone marrow biopsy, and a presumptive diagnosis of ET was given, supported by calreticulin (CALR) mutation detected. Low-dose aspirin and hydroxyurea were initiated, and platelets were at goal (<400 × 10^9^/L) two months later. Her history was further complicated by right central retinal vein occlusion in December 2016 during a period of poor compliance with cytoreductive therapy.

**Figure 1:**
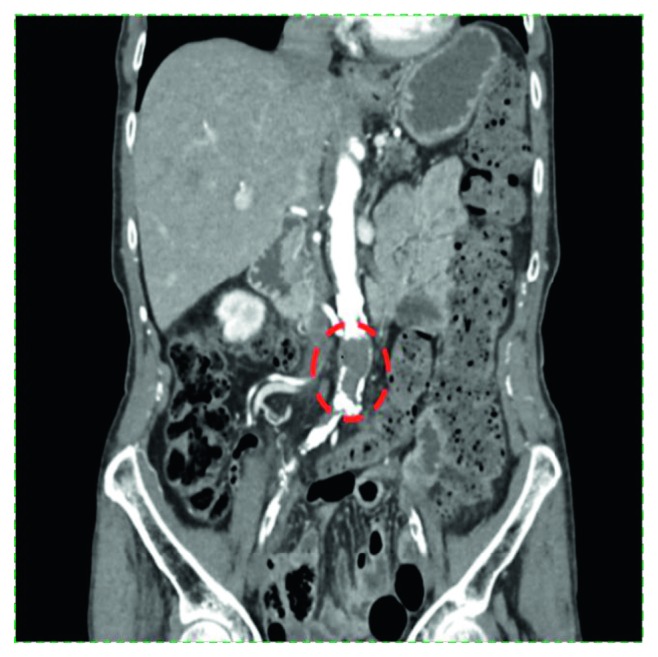
Computed tomography (CT) abdomen and pelvis demonstrating an occlusive thrombus in the abdominal aorta distal to the mesenteric artery. There is thrombus extension into the common iliac arteries with reconstitution of flow distally via collaterals.

In May 2018, she reported progressive fatigue and 15 lb unintentional weight loss over the preceding three months. Laboratory evaluation was notable for a macrocytic anemia and platelets at goal (WBC 6 × 10^9^/L; Hb 10 g/dL; MCV 101.7 fL; Plt 375 × 10^9^/L). She agreed to bone marrow evaluation which revealed a normocellular marrow with an increased number of enlarged and hyperlobated megakaryocytes and variable mild reticulin fibrosis (less than MF-1); blasts were not increased. Fluorescent in situ hybridization analysis for BCR/ABL, PDGFRA, PDGFRB, and FGFR1 was negative. Peripheral blood molecular analysis revealed a mutation in the calreticulin (CALR) gene; JAK2 V617F, JAK2 exon 12, and myeloproliferative leukemia virus (MPL) oncogene mutations were not identified. These findings confirmed a diagnosis of essential thrombocythemia and excluded myelofibrosis as the cause of her fatigue and weight loss. Further evaluation with CT scan of the chest, abdomen, and pelvis demonstrated atherosclerosis and completely occlusive thrombosis of the infrarenal aorta, extending bilaterally into the common iliac arteries, with reconstitution of flow distally via collaterals (Figure 1). There was no splenomegaly. Subsequent CT angiogram of the abdomen showed redemonstration of the aortic thrombus without occlusion of the mesenteric arteries, the celiac trunk, or the hepatic, splenic, and renal arteries.

The patient's only symptom was mild chronic claudication which had not changed in quality; she denied lower extremity swelling or skin mottling. She reported compliance with her prescribed medications for hypertension, hyperlipidemia, and ET. Lower extremity ultrasound and ankle brachial index were not significantly changed from prior studies. An evaluation for inflammatory disorders and thrombophilia, including erythrocyte sedimentation rate, C-reactive protein, anti-nuclear antibodies, anti-neutrophil cytoplasmic antibodies, lupus anticoagulant, anti-phospholipid antibodies, factor V Leiden mutation, prothrombin gene mutation, and protein C and protein S, was unremarkable.

In light of her very mild symptoms and good collateralization on imaging, she was offered medical management with surgical intervention reserved for treatment refractoriness or acute ischemia. Clopidogrel 75 mg daily was added to the aspirin 81 mg and hydroxyurea 1000 mg she was already taking. Additionally, she was started on cilostazol 100 mg twice daily by her vascular surgeon to treat her claudication. She reported initial improvement in her symptoms one month after starting therapy. She did not return for follow-up until February 2019 due to temporarily relocating out of the state. She had been noncompliant with therapy for ET. Her platelet count was 948 × 10^9^/L, and a CT scan showed no significant interval change in the occlusive thrombus of the infrarenal abdominal aorta and proximal common iliac arteries. She refused surgical intervention and was restarted on previous medical therapy.

## 3. Discussion

According to the 2016 World Health Organization, the diagnosis of ET can be made if the following major criteria are met: (1) platelet count ≥ 450 × 10^9^/L; (2) increased numbers of enlarged and hyperlobated mature megakaryocytes in the bone marrow without left shift and no more than grade 1 reticulin fibrosis; (3) not meeting criteria for other myeloproliferative neoplasms, myelodysplastic syndrome, leukemia, or other myeloid disorders; (4) presence of JAK2, CALR, or MPL mutation. Alternatively, ET may be diagnosed if the first three major criteria are met, and another clonal marker is identified or reactive thrombocytosis is excluded [1].

In this case, a clinical diagnosis of ET was originally given based on progressive thrombocytosis, increased large platelets on peripheral blood smear, and history of retinal vein thrombosis. The characteristic morphology on bone marrow evaluation three years later, negative evaluation for iron deficiency, infection, or inflammatory disorder, and CALR mutation detected confirmed the diagnosis of essential thrombocythemia. CALR is mutated in approximately 70% of patients with JAK2-negative essential thrombocythemia. The detection of a CALR gene mutation helps distinguish this clonal disease from a benign reactive process [8, 9].

Occlusive thrombosis of the aorta is rare and typically associated with aneurysm, dissection, or severe atherosclerosis. Hypercoagulable disorders, malignancy, and myeloproliferative disorders are less frequently described causative or contributing etiologies [10]. While small and medium vessel thromboses frequently complicate ET, only a small number of case reports have described thrombosis of the aorta. As such, there is no evidence-based consensus on how to treat these patients.

Thrombosis in ET is a result of spontaneous activation and aggregation of hypersensitive platelets [11]. Histopathologic evaluations of arterial thrombi in ET have revealed platelet-rich clots with abundant von Willebrand factor (VWF) and very little fibrin [12]. This pathophysiology supports the efficacy of antiplatelet and cytoreductive therapies in preventing and treating thrombosis and microvascular circulation disturbances in ET. Thrombosis while the platelet count is normal supports the view that platelet count is not the sole factor responsible for the thrombosis and thus, medical therapy targeted at thrombin generation may be less effective, given the limited fibrin in these platelet-rich thrombi [13].

The goal of medical therapy in ET is to prevent thrombohemorrhagic events in high-risk patients or prevent growth and embolization in patients with an existing thrombus. This is commonly accomplished with low-dose aspirin, which inhibits platelet activation, and hydroxyurea, which decreases the clonal production of hypersensitive platelets. Of the few cases of ET-related aortic thrombosis that have been published, most were managed with a combination of surgical and pharmacologic therapies; however, successful medical management alone has been reported [3–7, 14].

Fang et al. reported complete resolution of an intra-aortic thrombus, confirmed by CT scan, in an ET patient three weeks after starting aspirin and hydroxyurea [5]. Additionally, Lorelli and Shepard reported resolution of abdominal pain in an elderly patient with ET-related thrombosis of the aorta, splenic infarction, and portal vein thrombosis treated with anegralide and systemic anticoagulation with warfarin [3]. In a patient with myelodysplastic syndrome and/or myeloproliferative disorder unclassifiable (MDS/MPD-U) complicated by myeloproliferative thrombocytosis and aortic thrombosis, treatment with low-dose aspirin, hydroxyurea, and ticlopidine, a platelet adenosine diphosphate (ADP) receptor P2Y_12_ inhibitor, resulted in complete resolution of the thrombus on CT scan after one week of therapy [15].

In our patient, we hypothesize that atherosclerotic narrowing of the aorta and iliac vessels created a high shear environment whereby hypersensitive platelets, characteristic of ET, were activated and formed a platelet-rich thrombus despite a normal platelet count and reported compliance with antiplatelet and cytoreductive therapies. Noncompliance with antiplatelet therapy, though, cannot be ruled out, and unfortunately, platelet function testing was unavailable at our institution at that time. Our patient was minimally symptomatic, and so, the chronicity of her aortic thrombosis is unclear. She had numerous collateral vessels identified on imaging which likely developed as a result of her longstanding peripheral arterial disease. These collaterals may have protected her from experiencing acute symptoms when the occlusive thrombosis occurred. There was no acute indication for surgery at her initial presentations, so we aimed to intensify her medical therapy.

Taking into account the pathophysiology of arterial thrombosis in ET, additional platelet directed therapies were chosen over systemic anticoagulation. Clopidogrel, an irreversible adenosine diphosphate (ADP) receptor P2Y_12_ inhibitor, was added to aspirin and hydroxyurea to increase platelet inhibition. The patient was also started on a third antiplatelet agent: cilostazol, a phosphodiesterase type 3 inhibitor (PDE_3_), by her vascular surgeon to improve claudication. PDE_3_ inhibition leads to a cyclic AMP-dependent increase in protein kinase A (PKA) which prevents smooth muscle contraction resulting in vasodilation [16]. PKA also directly inhibits platelet activation and aggregation by a mechanism that remains poorly understood [17]. Though it has not been studied in ET, the safety and efficacy of this triple antiplatelet combination is supported by studies in other vascular diseases [18, 19]. Unfortunately, we could not assess the efficacy of this therapy in our patient due to poor compliance.

In conclusion, aortic thrombosis is a rare complication of ET and pharmacologic therapy alone may be appropriate in patients without acute surgical indications or who are poor surgical candidates. In patients who develop large vessel thrombosis on first-line therapies, additional platelet-directed therapies may be effective. This case also underscores the importance of managing cardiovascular comorbidities which increase thrombosis risk in patients with ET.
